# Transthyretin familial amyloid polyneuropathy (TTR-FAP) in Mallorca: a comparison between late- and early-onset disease

**DOI:** 10.1186/1750-1172-10-S1-P29

**Published:** 2015-11-02

**Authors:** Manuel Raya-Cruz, Juan Buades-Reines, Cristina Gallego-Lezaun, Ignacio Ferullo, Tomas Ripoll-Vera, Mercedes Uson-Martín, Hernan Andreu-Serra

**Affiliations:** 1 Son Llàtzer Hospital, Internal Medicine, 07198, Palma de Mallorca, Spain; 2 Son Llàtzer Hospital, Cardiology, 07198, Palma de Mallorca, Spain; 3 Son Llàtzer Hospital, Neurology, 07198, Palma de Mallorca, Spain; 4 Son Llàtzer Hospital, Digestive, 07198, Palma de Mallorca, Spain

## Background

The age of onset (AO) of familial transthyretin-mediated amyloidosis with polyneuropathy (TTR-FAP) is known to vary between populations, with differing characteristics reported according to AO in endemic/non-endemic foci.

## Methods

This was a retrospective study of patients with early AO (<50 years) and late AO (≥50 years) TTR-FAP at our community center in Mallorca. Data were collected on patient demographics, clinical disease manifestation, and physical symptoms.

## Results

A total of 95 patients were analyzed, with mean follow-up of 9.39 years from diagnosis. The early AO group included 53 patients (33 male) and the late AO group included 42 patients (21 male). Neurologic involvement was the most common initial symptom, although was significantly more frequent in the late AO versus early AO group (p=0.015). Autonomic involvement was observed in 26% of patients in the early AO group, but was rarely observed in the late AO group (5%). During follow-up, cardiologic symptoms, renal involvement, and ophthalmologic symptoms were significantly more common in the late AO group (p<0.05).

## Conclusions

This retrospective study demonstrates the variation in disease presentation and progression according to AO of TTR-FAP at our Mallorcan center. These data will inform diagnosis and monitoring of disease, and guide effective treatment choices.

